# Spatial and temporal patterns in Canadian COVID-19 crowdfunding campaigns

**DOI:** 10.1371/journal.pone.0256204

**Published:** 2021-08-23

**Authors:** Matthew McKitrick, Nadine Schuurman, Valorie A. Crooks, Jeremy Snyder

**Affiliations:** 1 Department of Geography, Simon Fraser University, Burnaby, British Columbia, Canada; 2 Faculty of Health Science, Simon Fraser University, Burnaby, British Columbia, Canada; University of Toronto, CANADA

## Abstract

Online charitable crowdfunding has become an increasingly prevalent way for Canadians to deal with costs that they would otherwise not be able to shoulder on their own. With the onset of COVID-19 and related lockdown measures, there is evidence of a surge in crowdfunding use relating to the pandemic. This study gathered, classified, and analysed Canadian crowdfunding campaigns created in response to COVID-19 from GoFundMe.com, a popular crowdfunding platform. Spatio-temporal analysis of classified campaigns allowed for observation of emergent trends in the distribution of pandemic-related need incidence and financial support throughout the pandemic. Campaigns raising money on behalf of established charities were the most common in the sample, and accounted for the greatest portion of funding raised, while campaigns for businesses made up a small proportion. Dense metropolitan areas accounted for the vast majority of campaign locations, and total sample funding was disproportionately raised by campaigners in Ontario and British Columbia.

## Introduction

In times of crisis, charitable crowdfunding serves as a popular method to connect those willing to give with those in need. From the half-billion dollars raised by the American Red Cross on behalf those impacted by the 2010 Haiti earthquake [1], to concerts raising money to fight SARS [2], and even to donations made at grocery store checkouts, crowdfunding initiatives funded by everyday people have been known to raise generous sums when the need arises. Even though these crowdfunding initiatives manifest in different forms, their unifying aspect is that they are charitable in nature, and on behalf of those in need as opposed to equity-based crowdfunding which raises money for the launch of a business venture.

In recent years charitable crowdfunding has expanded into the online space, allowing for individual people or groups to host ’campaigns’ on behalf of their particular cause in hopes of raising money directly from people in their personal network and beyond. GoFundMe, the largest of these platforms, has raised over $9 billion worldwide for campaigns on their platform since its launch in 2010, and in 2018 mediated roughly 90% of U.S. and 80% of global charitable crowdfunding dollars [3, 4]. GoFundMe and similar platforms host campaigns and allow campaigners to include text, images, videos, and periodic updates on the progress of their campaigns. This new medium of charitable crowdfunding has created a space for those with nowhere else to turn to solicit for financial assistance, and has resulted in the fulfillment of many campaign funding goals for worthy causes.

In addition to being more accessible to most people, the popularity of online crowdfunding platforms has also allowed researchers greater access to data on the topic, and thus more opportunity to analyze and investigate the manifestations of online charitable crowdfunding at large. Given the wide variety of platforms and absence of sanctioned access to this data, a diverse set of methods has been utilized in the literature to gather, analyze, and describe the various dimensions of online crowdfunding. Crowdfunding at large is investigated through the analysis of campaigns created by people seeking financial assistance associated with a need demonstrated by the content of the campaign, which are often manually searched and catalogued by researchers to develop a cohort pertaining to a specific topic from which conclusions can be drawn [5–7]. In contrast to work investigating the qualitative trends in a small number of highly specific campaigns, other research has utilized automated collection mechanisms like web crawlers to gather large cohorts of campaigns from which summative statistics can be generated [8–11]. Because campaigns include a mixture of both quantitative and qualitative information, research often incorporated a blend of methods and results which describe both these dimensions.

In light of the expanding body of research on the topic, many researchers have been critical of the ethical and societal implications of this new mode of donation based crowdfunding, and have increasingly found that it exacerbates inequality by awarding crowdfunding dollars not by relative need, but by ability to appeal to an audience through mastery of digital media and media literacy [12, 13]. Rather than alleviating societal inequities by allowing anyone to create a campaign, online donation-based crowdfunding has come under scrutiny for potentially perpetuating imbalances, replicating existing inequities in race, gender, and socio-economic status by favouring those with the means to create successful campaigns rather than those most severely in need [11, 14]. Geographic-oriented research in this domain similarly found evidence of spatial inequities, where communities in urban areas vastly outperformed their rural counterparts, bringing in drastically higher volumes of funding and support through crowdfunding platforms even when accounting for relative population densities [15]. Nevertheless, GoFundMe is a clear leader in the charitable crowdfunding market, and nothing has demonstrated this more clearly than the incredible volume of campaign creation and charitable donations in response to the COVID-19 pandemic.

The COVID-19 pandemic has been another crisis where individual people and groups have turned to crowdfunding for help and to aid others. Between March 1 and August 31, 2020, over $625 million was raised globally across 150,000 campaigns created to support causes related to the COVID-19 pandemic; in the US, approximately 60% of pandemic-related campaigns were created to support small businesses and those dealing with unemployment [16]. These figures show not only the impacts of the current crisis, the immense need it has generated, and the generosity and situational awareness of those still able to donate, but also the prevalence of GoFundMe and online charitable crowdfunding in general as a significant means with which society addresses economic hardship.

Canada has been no exception to the economic hardship caused by the COVID-19 pandemic. From February to April 2020, about 5.5 million Canadian workers had their employment situation negatively affected by the economic shutdowns, and in the proceeding period from May to September 2020, over 8.75 million unique applications were made to the Canada Emergency Response Benefit (CERB), equating to over $76 billion in funds being dispersed to Canadians in just 6 months [17, 18]. As a result, many Canadians have turned to crowdfunding to address the needs created by the COVID-19 pandemic and efforts to mitigate its spread.

In this article we provide a snapshot in time of COVID-19 related Canadian crowdfunding campaigns during the first 6 months of the pandemic. Specifically, the spatio-temporal characteristics of these campaigns are analyzed and visualized to show the patterns of campaign creation and success throughout Canada between January and June 2020. The aggregation and analysis of this data creates an opportunity to understand not only the needs that emerged in Canada as a result of the pandemic, but also where and when they arose relative to significant events in the pandemic timeline. This analysis is original in its spatio-temporal focus, using a map and several figures to visualize where in Canada COVID-19-related campaigns were created, when campaign creation for specific pandemic-related needs were most prevalent, and how these factors relate to campaign fundraising success.

## Methods

Since campaign data are not publicly available for bulk download, it must be gathered using algorithms to ’scrape’ data directly from the website if large volumes of data (too large to manually copy) are desired. This method of data collection has previously been used to target and gather campaigns raising money for a specific purpose, which are then quantitatively analyzed to examine the qualities of these campaigns. For example, Duynhoven et al. used scraped campaigns to analyze the spatial trends in Canadian crowdfunding campaigns for cancer [15], and more recently scraped campaigns have been used to examine the quantitative trends of crowdfunding for COVID-19 at large [19, 20].

The focus of this research is to explore the characteristics of Canadian crowdfunding campaigns created for reasons related to the COVID-19 pandemic. We gathered campaigns from only one platform, GoFundMe, due to its popularity, broad user appeal, and because combining campaigns across platforms can bring out platform-specific effects in the data [13]. The exploratory research design uses a broad analytic focus within a specific, robust sample, in search of emergent properties rather than the answer to a specific research question. The value of interpretation and discussion of campaign characteristics lies not in sample completeness, but in sample size and content. The scraped campaigns, as is common among scraped web datasets in general, do not form a probabilistic sample, and therefore are not statistically generalizable to the space of crowdfunding at large. Instead, the quantity of gathered campaigns and the specificity of their purpose allows for the examination of trends and dynamics between variables in the sample.

Crowdfunding campaigns were scraped from www.gofundme.com using a Python algorithm. The scraper gathered all of the campaigns resulting from searching "Canada" AND "COVID-19" via the GoFundMe search bar on June 30th, 2020, which amounted to 915 search results in total, each of which were saved to a local database. Four reviewers then examined a random sample of 100 of these campaigns, twice. Based on this examination and subsequent reviewer discussions, 6 content categorizations were developed to describe the underlying funding motivations present in the campaign sample. Shown in Table 1, these content categorizations were decided upon after extensive reviewer discussion surrounding the content of both the title and description of each campaign in their respective sample.

**Table 1:** Content categorizations used to describe the funding motivation present in the collected campaigns.

In support of a(n) ____________ in relation to the COVID-19 pandemic:	Description
	Funding Requested to…
1) Canadian Business	Help a small business dealing with lockdown-related closure or general loss of income during the pandemic.
2) Canadian Charity	Be donated to a formal charity for charitable purposes within For example: Canada Food Bank, Canadian United Way.
3) International Charity	Be donated to a formal charitable organization for charitable purposes outside of Canada. For example: UNICEF.
4) Purchase / Manufacture of Personal Protective Equipment (PPE)	Help with the purchase or creation of Personal Protective Equipment, particularly PPE that is thought to shield against COVID-19, such as face masks (N95, Surgical, Cloth), face shields, plexiglass barriers, and other such equipment.
5) Family Reunification	Help with airfare and other travel costs associated with reuniting family members and pets stranded because of COVID-19 related border closures and flight cancellations.
6) Personal Need	Directly help people who are experiencing hardship, financial or otherwise, due to the pandemic.

Each campaign in the sample was assigned one of these content descriptors to enable quantitative analysis of the pandemic-related funding motivations in Canadian COVID-19 crowdfunding campaigns. These categorizations were developed by analysis and discussion of random campaigns from the sample.

Campaign categorizations were generated as a result of two collaborative discussion sessions held after each author reviewed a subsample of campaigns. During the discussions, authors promulgated the main themes of their subsamples and drew from extensive previous experience reviewing crowdfunding campaigns to develop a collaborative content taxonomy that included the prominent categories of campaigns in the sample while simultaneously narrowing down the campaign types to 6 specific areas that aptly described the entire sample when assigned appropriately. After the categories were developed, a single reviewer analyzed each campaign, removing those campaigns that were either not created in Canada, were created outside the prescribed study period from January—June 2020, or were not directly motivated by COVID-19 related circumstances. The start of the time period (January 2020) was chosen as a safe estimate for the earliest possible time for which COVID-19 campaigns may have been created, while the period end date (June 2020) was chosen so as to capture the campaigns from the first 6 months of the pandemic to identify emerging crowdfunding responses to COVID-19.

During this process the reviewer also assigned one of the six type descriptors to each campaign in the sample. At this stage, each campaign consisted of seven attributes, each attribute corresponding to a data column: Campaign title, Description, Date of Creation, Funding Dollars Raised, Funding Dollars Requested, Location, and Type, shown in Table 2. The location attributes were gathered in the form of place names, which were subsequently geocoded using QGIS’s integrated geocode function to allow for the spatial analysis component. Table 2 serves to exemplify a standard case of a campaign scraped by the Python web scraper, where each row contains a single campaign and each column contains an a campaign attribute derived from the HTML webpage on www.gofundme.com for each individual campaign, which are the subject of the following analysis.

**Table 2:** Campaign dataset headers and example campaign entry.

Title	Description	Date Created	Funding Raised (CAD$)	Funding Requested	Location	Type
PPE for Frontline Workers	Donations towards the purchase of PPE for frontline workers in Toronto hospitals	05-01-2020	$51,453	$100,000	Toronto, Ontario	PPE

Campaigns relating to the pandemic were scraped from Gofundme.com in the format shown here. The ‘Type’ column was appended to reflect the type of need portrayed in the title and description. Location and date created values acted as space and time variables, respectively. The funding requested column shows how much money was raised by the campaign from the date it was created until June 15th, 2020.

## Results

A total of 915 campaigns were initially scraped from www.gofundme.com, each of which were examined for inclusion criteria; 342 campaigns were removed during this process. The remaining 573 campaigns were analyzed spatially, temporally, monetarily, and according to content, corresponding with the variables of campaigner location, date of campaign creation, funding dollars raised per campaign, and campaign funding, respectively. The main reasons why campaigns were not included in the analysis was due to invalid campaign content (not directly related to COVID-19), invalid location (not in Canada or not in a specific Province or Territory), or because the location was unable to be geocoded.

Table 3 shows a breakdown of these variables. The following 7 figures will visualize the trends seen in the scraped campaign across these 4 dimensions.

**Table 3:** Campaign study variables derived from scraped campaign data.

Variable	Description
Campaigner Location	The listed location of the campaign, usually at the municipal level.
Campaign Date of Creation	Date that the campaign was created on.
Campaign Dollars Raised	Funding dollars raised by the campaign (CAD$).
Campaign Type	Underlying type of campaign fundraising need (See Table 1).

**Fig 1:**
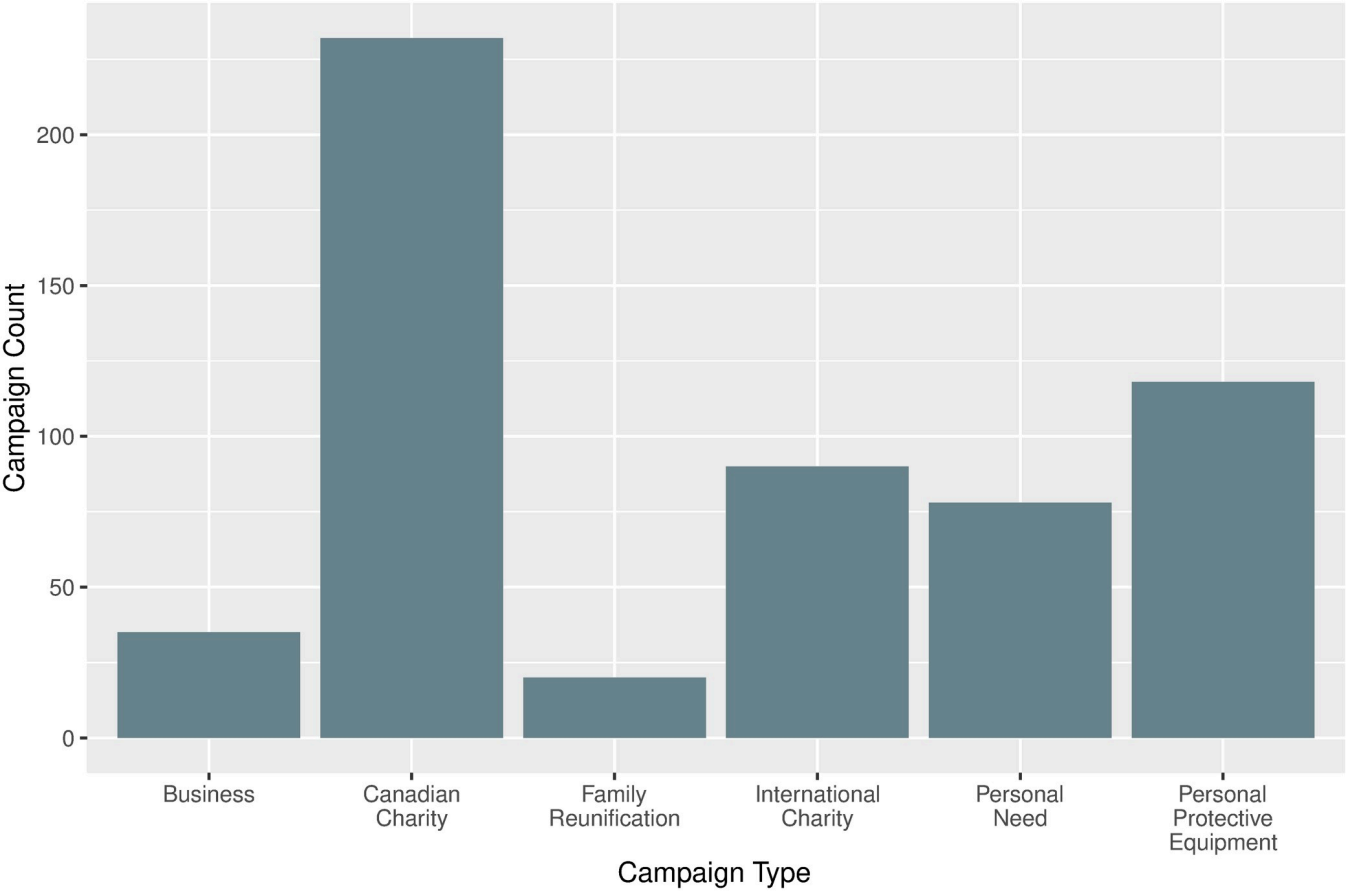
Campaign count by type. A total of 573 campaigns were categorized by the type of need they addressed. The figure shows the type of need demonstrated by the gathered campaigns relating to COVID-19 from January 1st to June 15th, 2020. For a description of each ’Campaign Type’, refer to Table 1.

First, the campaigns are counted and shown by type (Fig 1). Campaigns created by, or on behalf of charities were the most numerous, accounting for 232 of the 573 total campaigns. The majority of these campaigns were created by people on behalf of established charities to raise money in their particular group, while a comparatively small number of campaigns in this category were created by the charitable organizations themselves. This latter group was clearly identifiable, as the campaigns were created by the verified user accounts of the charity and usually had much larger levels of donations. Campaigns for PPE [118], international charities [90], and general personal need [78] accounted for roughly half of the total distribution, while campaigns for private businesses and family reunification together accounted for about 10%.

**Fig 2:**
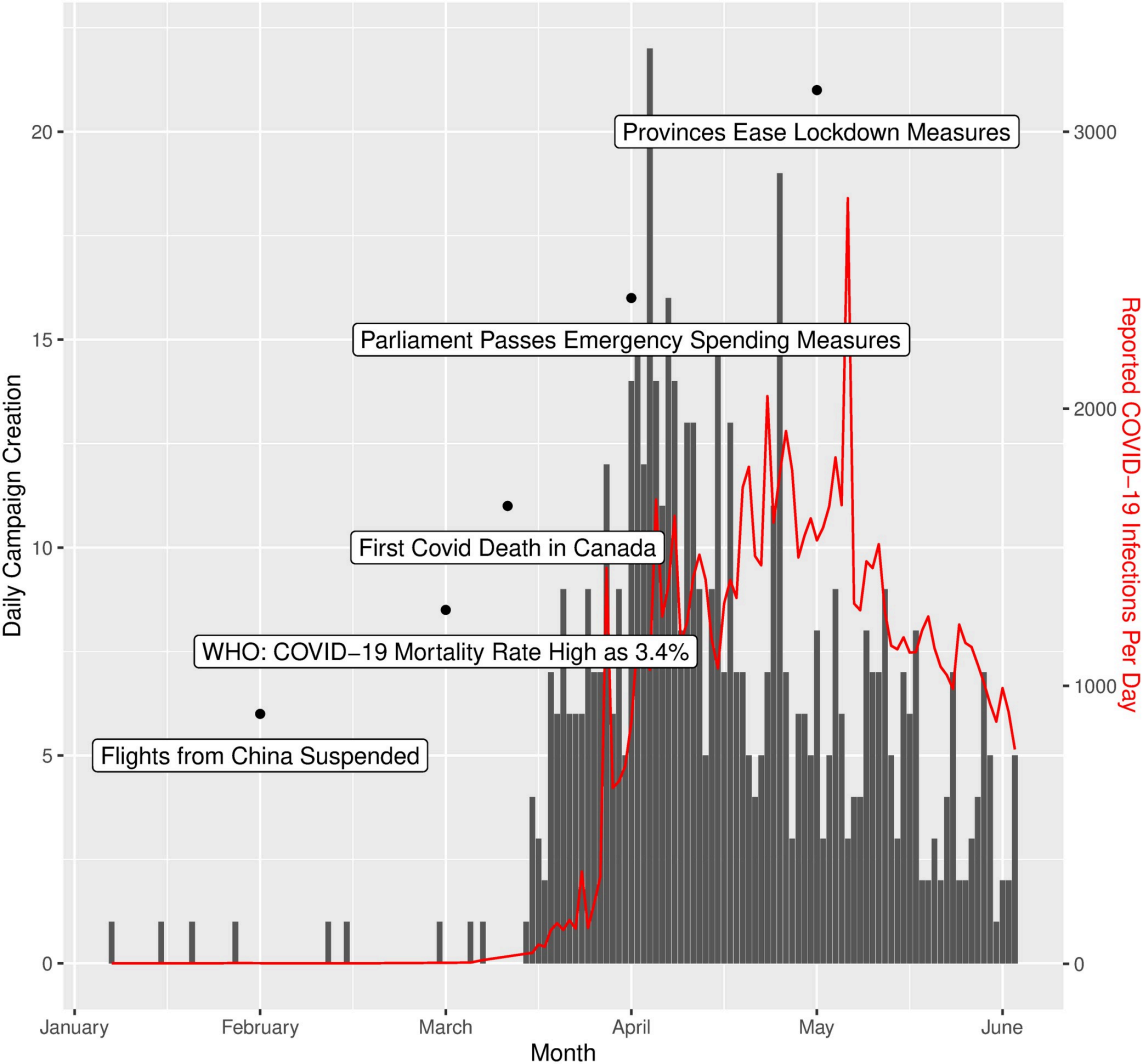
Campaigns created per day vs. Canadian COVID-19 timeline from January 1st to June 15th. The volume of campaigns created per day are shown in grey bars, the daily number of new COVID-19 cases is shown with the red line, and various COVID-19 related events are shown with labels corresponding to the date of the black dot above each of them.

Next, the count of COVID-19 related campaigns created per day was plotted against the reported number of daily new confirmed COVID-19 cases in Canada, with major related news events superimposed in series (Fig 2). The number of campaigns per day, shown as the grey bars, peaks in early April, as the number of daily new cases surpassed 1000 per day, but tapered off shortly after and remained low into June. The number of new cases peaked almost a month after the apex of campaign creation, and steadily declined into June. The majority of campaigns (68%) were created in the six weeks from mid-March through the end of April, in roughly the same period of time as between the first COVID-19 death in Canada and the initial easing of the lockdown measures.

**Fig 3:**
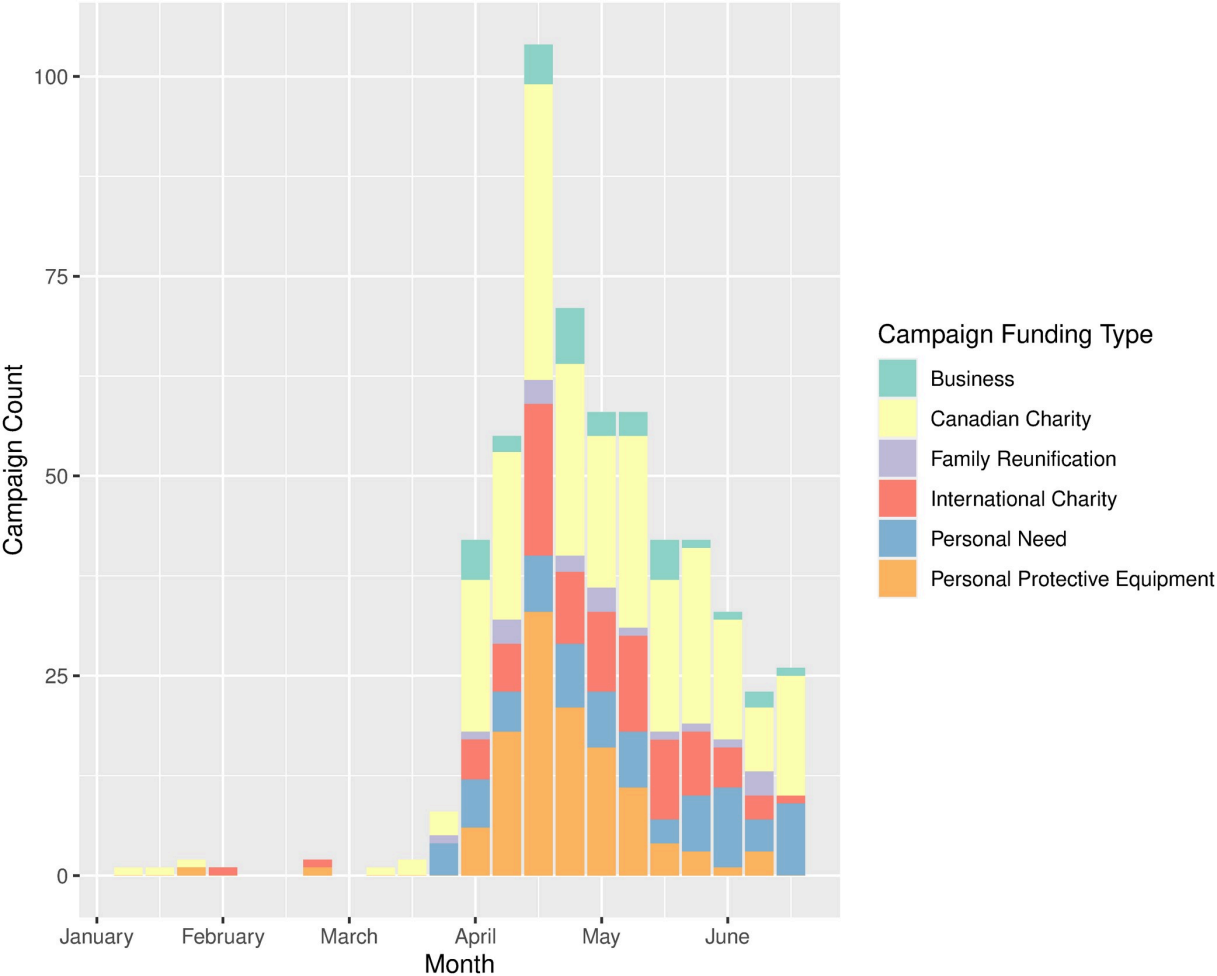
Campaign class type distribution per week of the study period from January to June 2020. Shows the trends in weekly campaign creation, stratified by campaign type, across the 22 week period within which all scraped campaigns were created.

Campaign types were then plotted as a time series by date of creation to show the temporal distribution of need as it arose during the pandemic (Fig 3). Campaigns created for the purposes of funding a Canadian charity made up the largest proportion of newly created campaigns for the majority of weeks in the study period. Campaigns for the purchase or manufacture of PPE peaked in mid-April but tapered off and were not a significant proportion into May. Campaigns for family reunification in Canada from abroad and Canadian businesses were sporadic and minimal for most weeks, while campaigns for personal need were steady throughout. Finally, campaigns for international charities peaked in early April and occupied a consistent proportion of weekly campaigns until June.

**Fig 4:**
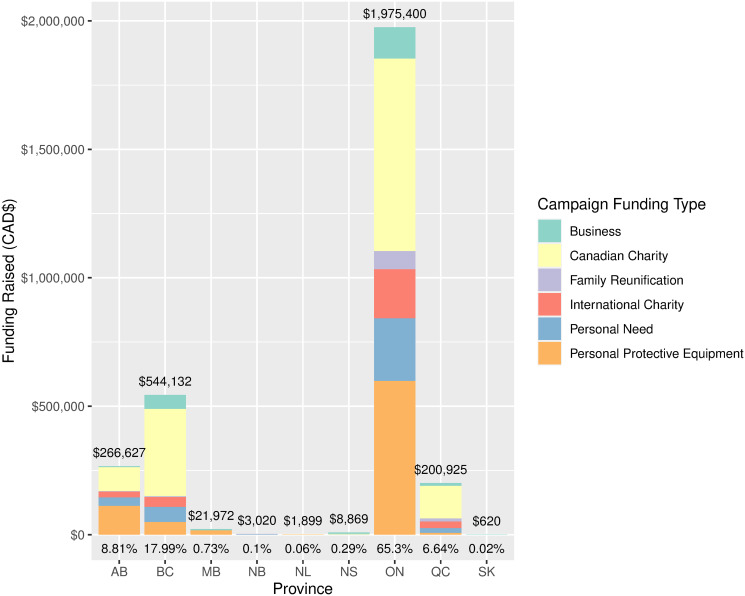
Total funding dollars raised per province from the 573 campaign sample by campaign type. Ontario received the vast majority of total funding (65.3%), followed by BC (17.99%), Alberta (8.81%), Quebec (6.64%), and the remaining provinces accounting for about 1% of total donations. Funding distributions per campaign type within the provinces showed similar patterns to the distribution of campaigns by type at the national level.

Fig 4 shows the provincial distribution of donations to campaigns in the sample, again stratified by campaign type. Ontario had by far the most donations, accounting for 64.3% of total funding in the sample. British Columbia, Alberta, and Quebec followed with 17%, 10%, and 7% of funding, respectively, with the remaining provinces accounting for the last 1%. The distribution of funding per type in each province remained similar to that breakdown at the national level, with campaigns for Canadian charities accounting for a considerable proportion in each province, and when including PPE account for the vast majority of funding dollars raised by campaigns in the sample. None of the Territories had any campaigns, and Manitoba (MB), New Brunswick (NB), Newfoundland and Labrador (NL), Nova Scotia (NS), and Saskatchewan (SK) accounted for about 1% of total funding.

**Fig 5:**
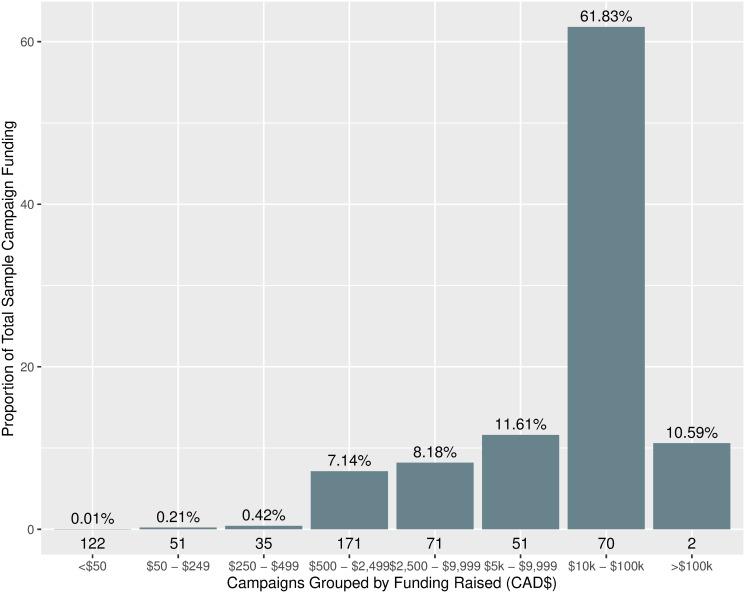
Funding raised by campaigns grouped by total dollars raised. Campaigns were assigned a grouping based on the funding raised relative to the ranges shown on the x-axis. The counts of how many campaigns were included in each group are shown underneath each corresponding bar in the chart. The proportion of funding raised by campaigns in each funding range grouping is shown on the y-axis, and is relative to the total dollars raised by all campaigns in the sample ($3,024,967).

Fig 5 shows the proportion of total dollars raised that each campaign funding group accounted for. Campaigns were grouped by how much money they had raised into bins shown along the X-axis of Fig 5. The $500–2500 group accounted for the most campaigns [171], but the $10,000–$100,000 raised group accounted for the majority of funding dollars raised, with 61.83%. There were only 2 campaigns that raised more than $100,000; between them they accounted for 10.59% of all funding dollars raised by campaigns in the sample. Although there were 122 campaigns that raised between $0–50, these campaigns accounted for less than 0.01% of total dollars raised, and 86 campaigns raising between $50–250 and $250–500 together accounted for just 0.63% of all funding.

**Fig 6:**
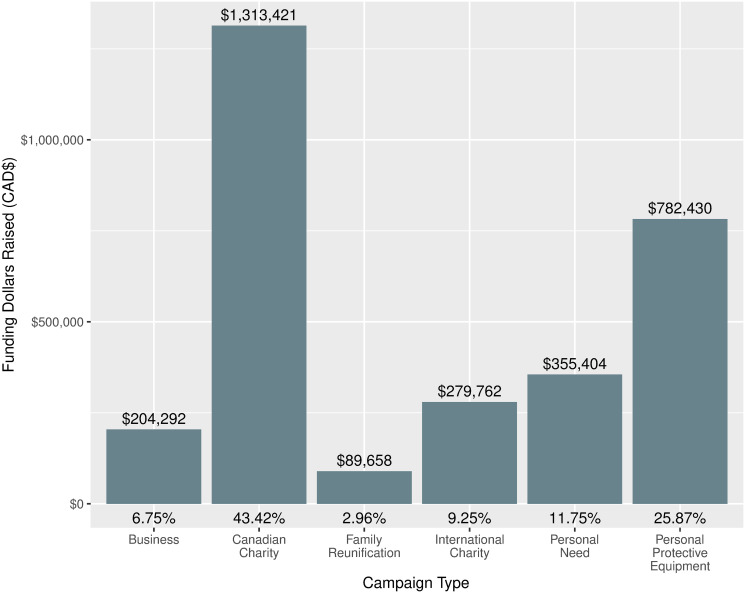
Dollars and proportion of total funding raised by campaign type. The dollar value atop each bar indicates how many dollars were raised by campaigns of the corresponding type, cumulatively. The percentage figure below the bar shows what proportion of total dollars raised by campaigns in the sample each dollar amount corresponds to *(N dollars raised = $3*,*024*,*967)*.

Fig 6 shows funding raised by campaign type. Again, campaigns for Canadian Charities and PPE were representative of the majority (>70%) of funding, over $2 million (CAD). The distributions here are similar to those shown stratified by week and count of campaign type, with campaigns for businesses and family reunification accounting for about 10%, and those for Personal Need and International Charities the remaining 20%.

**Fig 7:**
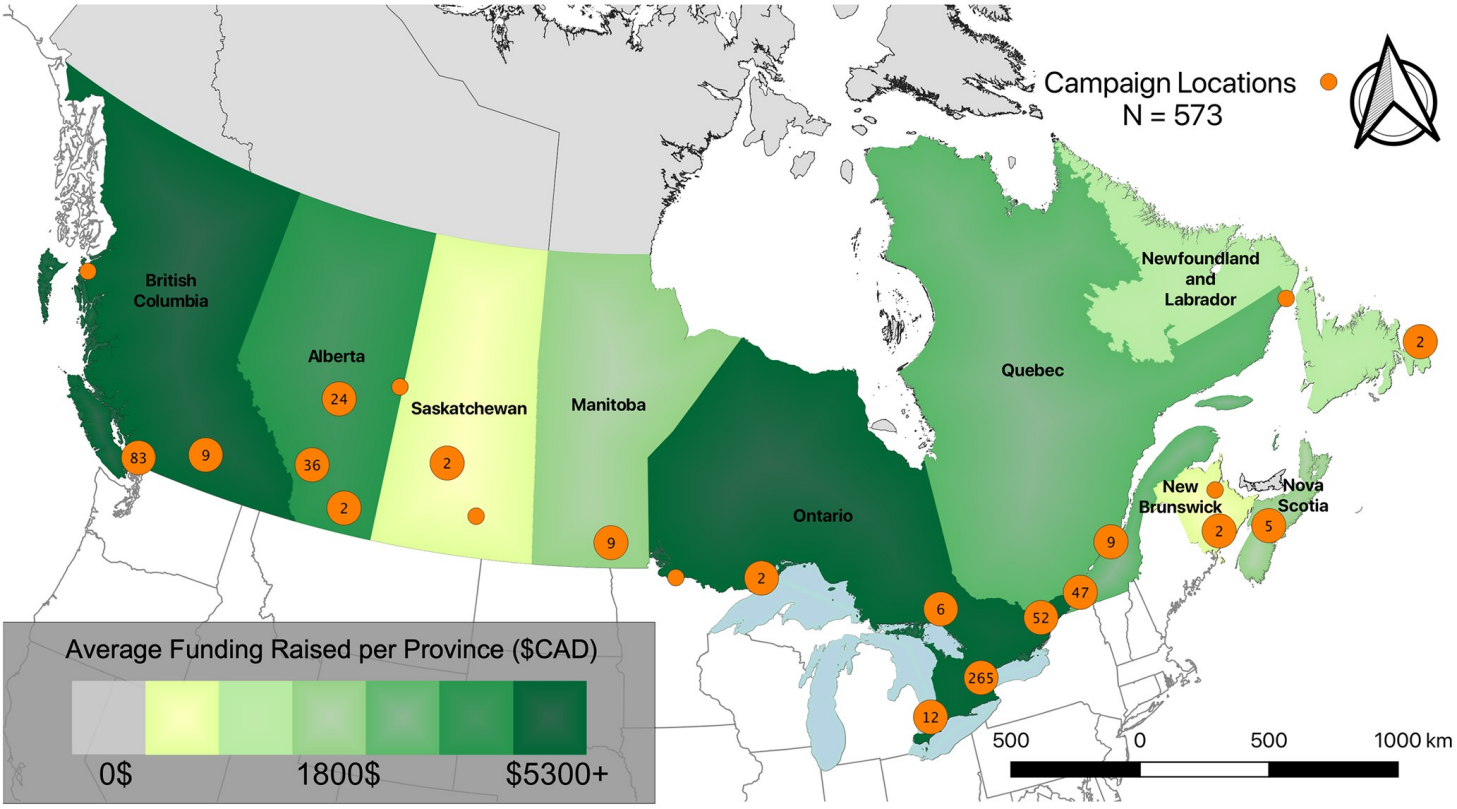
Spatial distribution of campaigns in Canada and average campaign funding per province. Most campaigns in Canada were found to be surrounding the country’s major metropolitan areas, like Toronto, Vancouver, Montreal, and Ottawa. Ontario showed the highest average donation, Saskatchewan the lowest.

Finally, Fig 7 shows the spatial distributions of campaigners across Canada superimposed over color-coded provinces depicting the average level of funding received by campaigns in each province. The dense, urban areas of Canada like Toronto, Vancouver, Montreal, Ottawa, and Calgary show high concentrations of campaigners, with few campaigners shown in predominately rural or Northern areas of the country. Ontario and British Columbia (BC) showed the highest average campaign funding raised, followed by Alberta and Quebec. No campaigns created in the Territories or Prince Edward Island were included in the study sample; Saskatchewan, Newfoundland and Labrador, and New Brunswick showed fewer than 5 campaigns each, Manitoba and Nova Scotia had less than 10 each.

## Discussion

Distributions of campaigns according to campaign funding objective showed similarities with other analyses of COVID-19 campaigns, specifically the prevalence of campaigns for charities (international or local), PPE, businesses, and general areas of personal need (Elmer et al. 2020). However, the campaigns in the present study showed a much higher proportion of campaigns for charitable organizations (accounting for over 50% of campaigns in the sample), and more particularly campaigns for charities within the same country/community (as opposed to charities working internationally). Campaigns for PPE were also very common in both studies [19] likely due to the widespread shortages experienced worldwide at the onset of the pandemic [21].

Campaigns for businesses struggling as a result of the pandemic and related lockdown measures were surprisingly few in number and in dollars raised, accounting for only about 6% of both campaigns in the sample and funding dollars raised despite widespread coverage of the fact that significant numbers of small businesses were and are facing bankruptcy and closure [22, 23]. This could be due to the time period within which these campaigns were gathered, as businesses may have been able to stay solvent during the early days of the pandemic, surviving on cash reserves and government aid. A future analysis of campaigns from later periods in the pandemic could reveal a greater prevalence of business-related campaigns, as dampened revenues and lockdown measures persist in Canada.

Campaigns for personal need in Canada made up about 14% of campaigns in the sample and almost 12% of dollars raised ($355,404), which, while a significant amount of money, was lower than expected relative to the other categories given the broad definition of ’personal need’ used here and the wide variety of potential personal needs that can arise during a pandemic. For example, a previous campaign analysis found that campaigns for funerals, family/friend support, and food/supplies (all included in the present ’personal need’ category) together made up 31% of sample COVID-19 related campaigns [19]. Given the extensive aid offered by the Canadian government to Canadians in the amount of $2,000 monthly cheques [17], it could be that the demand for crowdfunding dollars on behalf of individual Canadians in need as a result of the pandemic is lower in the present study due to the specifically Canadian sample, as opposed to the non-country specific sample of campaigns gathered by Elmer et al. (2020).

Campaigns for family reunification, although relatively few in comparison to other categories, still raised almost $90,000 in total, and were included to demonstrate this distinct type of need. As opposed to campaigns for businesses, it seems likely that campaigns for family reunification were prevalent at the beginning of the pandemic due to the abrupt nature of border closures and orders for Canadians to return home from abroad, but tapered off as everyone who needed to arrive home had already done so, demonstrated by the absence of campaigns in this category from the last week of the study period (Fig 3).

Temporal trends in campaign creation showed that the initial spike in campaigns coincided precisely with the first surge in COVID-19 cases in Canada (Fig 2). Campaigns for charities and PPE dominated these early weeks. This shows the reactionary crowdfunding response of Canadians as it became clear that COVID-19 was to become a serious societal crisis, and how digital donation-based crowdfunding acted as a primary medium for Canadians to manage the crisis, whether by donating, or by campaigning for themselves or on behalf of others. Similar trends were seen in American crowdfunding, where creation of COVID-19 related campaigns coincided temporally with increases in detected COVID-19 cases [20].

Spatial analysis of campaigns showed a clear urban-rural divide in Canada, where the vast majority of campaigns were created in densely populated, urban areas. This is undoubtedly due to asymmetries in Canada’s population distribution, as demonstrated by the vast majority of funding dollars also being raised in the most populous Provinces, however it could also be due to lower levels of access to communication technologies in rural communities or to the strength of voluntary or informal care sectors in rural communities were residents look inwards to friends and neighbours for support rather than outwards to society at large [15].

This study focused on analyzing the spatio-temporal patterns in crowdsourced data from GoFundMe.com. As such, it is differentiated from studies using similar data with a focus on inferential statistics [11]. Studies like Kenworthy et al. (2020) used a randomized sample of campaign data derived from an extremely large dataset (>165,000 campaigns) to draw and extrapolate inferential conclusions about crowdfunding users, campaign characteristics, and crowdfunding success at the national level [11]. Analyses such as this, which favour statistical significance and inference instead of considering spatio-temporal variables, are therefore distinct from the purview of the present paper because conclusions are drawn from aspatial factors. However, clearly both approaches reveal patterns in the data and are complementary.

### Limitations

The present study acknowledges limitations to the methods used in gathering and analyzing crowdfunding campaigns. First, it is entirely possible that the search protocol over/under exaggerated certain types of campaigns, or entirely missed campaigns that would have otherwise met inclusion criteria. For example, the large number of campaigns on behalf of Canadian charities included in the sample could be due in part to the utilization of ’Canada’ in the GoFundMe search, which would favour campaigns for organizations like the ’Canada Food Bank’ or ’Canadian United Way’ as the word ’Canada’ is explicitly used in the title and campaign description. In contrast, campaigns for an individual person looking for donations from their local network would perhaps not include the word ’Canada’, even if the campaign was in Canada because it would be self-evident to those donating. Given the nature of the campaign data and that the only way to access it is through the front-end of the website (as opposed to having access to the back-end which would allow for more options when querying), it is impossible to say whether or not all the relevant campaigns were included. However, since all the campaigns included in the final analysis were individually examined and vetted for inclusion criteria, we can say that the sample represents a robust, though likely incomplete, collection of relevant campaigns.

Another limiting factor is that the sample here represents a snapshot of campaigns at the time they were harvested. This means that, although campaigns as far back as January 2020 were gathered, it is possible that many campaigns were created and then deleted before having a chance to be gathered. This could lead to an over representation of successful campaigns, as unsuccessful campaigns are rather more likely to be deleted after a short time [15]. This snapshot also only gathered information on the first 6 months of the pandemic; future studies could investigate the characteristics of crowdfunding campaigns during the latter portions of the pandemic to observe whether the same types of campaign characteristics persisted.

## Conclusion

Charitable crowdfunding has become an increasingly popular way for people to deal with unmanageable expenses, and the expenses incurred by many millions of Canadians over the last twelve months due to COVID-19 are no exception. Collection and analysis of COVID-19 related crowdfunding campaigns from GoFundMe revealed a significant number of Canadians who had turned to crowdfunding to help themselves personally, but more so on behalf of charitable organizations in Canada and abroad. The spatio-temporal approach allowed for identification of trends across Canada and across time with respect to COVID-19 and how the pandemic developed from its beginnings in February to the lull in cases and relaxing of restrictions in June. Funding and campaign need type were the primary dimensions of analysis, both of which showed clear patterns when analyzed across time and space in Canadian provinces, with the most pronounced numbers of campaigns and funding dollars raised being in BC and Ontario during the months of April and May. Campaign creation was also greatest in the period between when the initial lockdown measures were imposed and when the Canada Emergency Response Benefit was announced.

The study design in terms of campaign collection, classification, and analysis allowed for campaigns on the charitable crowdfunding website GoFundMe to be used as a proxy for understanding how these types of platforms facilitate need in times of crisis, as well as the specific areas of need in Canada that were revealed as a result of the pandemic. While other studies on COVID-19-crowdfunding have been published, our results demonstrate that spatio-temporal specificity allows for a nuanced study in how pandemic-related needs and crowdfunding change over time and react to events and policies in specific contexts. Future studies utilizing a similar collection protocol should aim to minimize the campaign selection bias in the collection process by performing multiple searches using utilizing multiple search terms across a period of weeks or months, to ensure the greatest possible number of relevant campaigns are collected.

## Supporting information

S1 Data (TAR)
